# 2^nd^ International External Quality Control Assessment for the Molecular Diagnosis of Dengue Infections

**DOI:** 10.1371/journal.pntd.0000833

**Published:** 2010-10-05

**Authors:** Cristina Domingo, Matthias Niedrig, Anette Teichmann, Marco Kaiser, Leonid Rumer, Richard G. Jarman, Oliver Donoso-Mantke

**Affiliations:** 1 Robert Koch Institute, Berlin, Germany; 2 United States Army Medical Component of the Armed Forces Research Institute of the Medical Sciences (USAMC-AFRIMS), Bangkok, Thailand; University of California, Berkeley, United States of America

## Abstract

**Background:**

Currently dengue viruses (DENV) pose an increasing threat to over 2.5 billion people in over 100 tropical and sub-tropical countries worldwide. International air travel is facilitating rapid global movement of DENV, increasing the risk of severe dengue epidemics by introducing different serotypes. Accurate diagnosis is critical for early initiation of preventive measures. Different reverse transcriptase PCR (RT-PCR) methods are available, which should be evaluated and standardized. Epidemiological and laboratory-based surveillance is required to monitor and guide dengue prevention and control programmes, i.e., by mosquito control or possible vaccination (as soon as an effective and safe vaccine becomes available).

**Objective:**

The purpose of the external quality assurance (EQA) study described is to assess the efficiency and accuracy of dengue molecular diagnosis methods applied by expert laboratories.

**Study Design:**

A panel of 12 human plasma samples was distributed and tested for DENV-specific RNA. The panel comprised 9 samples spiked with different DENV serotypes (DENV-1 to DENV-4), including 10-fold dilution series of DENV-1 and DENV-3. Two specificity controls consisted of a sample with a pool of 4 other flaviviruses and a sample with chikungunya virus. A negative control sample was also included.

**Results:**

Thirty-seven laboratories (from Europe, Middle East Asia, Asia, the Americas/Caribbean, and Africa) participated in this EQA study, and reports including 46 sets of results were returned. Performance among laboratories varied according to methodologies used. Only 5 (10.9%) data sets met all criteria with optimal performance, and 4 (8.7%) with acceptable performance, while 37 (80.4%) reported results showed the need for improvement regarding accomplishment of dengue molecular diagnosis. Failures were mainly due to lack of sensitivity and the presence of false positives.

**Conclusions:**

The EQA provides information on each laboratory's efficacy of RT-PCR techniques for dengue diagnosis and indicates for most laboratories an urgent need to improve sensitivity and specificity.

## Author Summary

Dengue viruses (DENV) are the most widespread arthropod-borne viruses which have shown an unexpected geographic expansion, as well as an increase in the number and severity of outbreaks in the last decades. In this context, the accurate diagnosis and reliable surveillance of dengue infections are essential. The laboratory diagnosis of dengue relies on the use of several methods detecting markers of DENV infection present in patient serum. Molecular diagnosis methods are usually rapid, sensitive, and simple when correctly standardized. Moreover, PCR-based diagnosis techniques are able to readily detect DENV during the acute phase of the disease and may assume an important role in dengue diagnosis and surveillance. Different reverse transcriptase PCR (RT-PCR) methods have been developed and are currently available and should be standardized in each laboratory to maintain high quality performance. In this work an External quality assessment (EQA) activity has been carried out to evaluate the accuracy and quality of laboratory data for the molecular diagnosis and surveillance of dengue, which involved worldwide dengue reference laboratories. In conclusion, RT-PCR techniques for dengue diagnosis applied by the participating laboratories demonstrated the need of further improvement in most laboratories.

## Introduction

Dengue viruses (DENV) are transmitted by *Aedes sp.* mosquitoes and are members of the *Flaviviridae* family, genus *Flavivirus*. DENV comprise four antigenically distinct serotypes (DENV1-4), which are genetically quite distinct. Infection with one DENV serotype leads to lifelong protection against homologous challenge, but only brief cross-protection against heterologous infection with a different serotype[1].

The four serotypes of DENV are the causative agents of one of the most important arthropod-borne viral diseases in terms of human morbidity and mortality. Several virus and host-specific factors have been suggested to correlate with severe disease outcomes, which are mostly associated with secondary infections with a heterologous serotype[2] and/or infections with more intrinsically virulent strains of the virus[3], [4].

Epidemic dengue fever (DF) and dengue haemorrhagic fever (DHF) have emerged as a public health problem in recent decades, with the development of hyperendemicity (different DENV serotypes overlapping in an spatiotemporal manner), in urban and periurban centres of many tropical and subtropical countries. Air travel is also facilitating the rapid global movement of DENV and increasing the risk of DHF through the introduction of different serotypes into new areas. Two and a half billion people live in areas where the disease is endemic, and 50–100 million cases of DF are estimated to occur every year, including 500,000 cases of the more severe illnesses, DHF and dengue shock syndrome (DSS)[5].

The laboratory diagnosis of dengue relies on the use of several methods detecting markers of DENV infection present in patient serum. Definitive diagnosis of dengue rests on the detection of the infective virus, virus-encoded antigens, viral genomic RNA, or virus-induced antibodies. PCR-based diagnosis techniques can be more sensitive than viral culture, since they are able to readily detect the dengue viruses during the acute phase of the disease, and may assume an important role in dengue diagnosis, since detection of IgM is not possible practically until defervescence[6]. Molecular diagnosis methods are usually rapid, sensitive, and simple when correctly standardized and can be used for genome detection in human clinical samples, biopsies, autopsy tissues, or mosquitoes. However, specimens and RNA must be carefully handled to avoid RNA degradation. Further, RT-PCR is highly sensitive to amplicon contamination, and without proper controls false-positive results may occur.

Different RT-PCR methods have been developed for detecting and typing DENV, which vary somewhat in terms of the amplified gene regions of the genome, in the ways they detect RT-PCR products, and the virus-typing procedures. RT-PCR methods for detecting dengue virus-infected *Aedes* mosquitoes in the field may serve as an additional early warning monitoring system for predicting dengue outbreaks [7]. Moreover, since a change in serotype would be particularly important in bringing about a surge in DF/DHF cases, molecular methods are further advantageous in detecting the specific circulating serotypes both in dengue-infected patients and in natural populations of mosquitoes.

Aiming at the evaluation of the accuracy and estimation of the quality of laboratory data for the molecular diagnosis and surveillance of dengue, we have realised an external quality assurance (EQA) activity which involved worldwide dengue reference laboratories.

## Materials and Methods

### Participants

Laboratories in Europe (n = 22), Middle-East Asia (n = 1), Asia (n = 8), Africa (n = 1), and the Americas/Caribbean (n = 5) participated in the EQA activity: Institut für Virologie, Philips Universität Marburg, Germany; Institut für Virologie, Universitätsklinikum Bonn, Germany; Institut für Virologie, Medizinische Universität Wien, Austria; CNR Associé des Arbovirus IRBA-Antenne Marseille-IMTSSA, France; Institut Pasteur, Centre de Réference des Arbovirus, Marseille, France; Centro Nacional de Microbiología, Instituto de Salud Carlos III, Madrid, Spain; Instituto Tecnológico “La Marañosa”, Madrid, Spain; Aristotle University of Thessaloniki, School of Medicine Department of Microbiology, Greece; Erasmus Medical Centre, Rotterdam, The Netherlands; Spiez Biology Laboratory, Spiez, Switzerland; Institut für Klinische Mikrobiologie und Immunologie, St. Gallen, Switzerland; University of Geneva, Laboratory of Virology, Geneva, Switzerland; Army Medical Research Center, Rome, Italy; Department of Infectious, Parasitic and Immunomediated Diseases, Instituto Superiore di Sanità, Rome, Italy; Instituto Nazionale per le Malattie Infettive “L. Spallanzani”, Rome, Italy; Laboratory of Vector-borne Infections, Cantacuzzino Institut, Bucarest, Romania; Instituut voor Tropische Geneeskunde, Centraal Laboratorium Klinische Biologie, Antwerpen, Belgium; Haartman Institute, University of Helsinki, Finland; Statens Serum Institut, Department of Virology, Copenhagen, Denmark; “Béla Johan” National Center for Epidemiology, Budapest, Hungary; Norwegian Institute of Public Health, Oslo, Norway; National Reference Laboratory for Arboviruses, National Institute of Public Health, Ostrava, Czech Republic; National Centre for Zoonotic Viruses, Tel Hashomer; Israel; Department of Laboratory Medicine, Tan Tock Senk Hospital, Singapore; Department of Molecular Medicine, National University Hospital, Singapore; Department of Pathology, Singapore General Hospital, Singapore; Environmental Health Institute, Singapore; Hospital for Tropical Diseases, Oxford University Clinical Research Unit, Ho Chi Minh City, Vietnam; Tropical Infectious Diseases and Education Center, University Malay, Kuala-Lumpur, Malaysia; Institut Pasteur, Cambodia; Special Pathogens Unit, NICD-NHLS, Sandringham, South Africa; Caribbean Epidemiology Center (CAREC), Port of Spain, Trinidad; Laboratorio de Flavivirus, Instituto Oswaldo Cruz, FIOCRUZ, Rio de Janeiro, Brazil; Centro de Investigación en Enfermedades Infecciosas, INER, Tlalpan, Mexico; Instituto Nacional de Salud, Bogotá, Colombia; Instituto Nacional de Enfermedades Virales Humanas Dr. Julio Maiztegui, Pergamino, Argentina.

The EQA was established and coordinated by the European Network for the Diagnostics of ‘Imported’ Viral Diseases-Collaborative Laboratory Response Network (ENIVD-CLRN) which has previously coordinated EQA studies [8], [9], [10].

### Specimen preparation and dispatch

Inactivated and stable DENV preparations were generated from cell culture (Vero B4) supernatants of the 4 dengue serotypes: DENV-1 VR344 (Thai 1958); DENV-2 VR345 (TH-36 strain); DENV-3 VR216 (H87 strain); and DENV-4 VR217 (H241 strain). Viral cell supernatants were inactivated by heating for 1 h at 56°C and gamma irradiation [25 kilogray (kGy)] to assure their non-infectivity.

Sample preparations were tested by an in-house real-time quantitative RT-PCR with a sensitivity threshold of 5 genome equivalents per reaction (GE)/rxn (100 GE/ml sample). Intra-assay and inter-assay variance of this method were determined as <3% and <4%, respectively. The inactivated supernatant viral load was estimated after heat inactivation and additionally after gamma irradiation. The inactivated supernatants were diluted in serum plasma to prepare a set of 9 positive samples which included 5 serial 10-fold dilution series of DENV-1 (7×10^5^ GE/ml to 70 GE/ml), and two DENV-3 dilutions (3×10^4^ GE/ml and 3×10^3^ GE/ml). DENV-2 and DENV-4 inactivated supernatants were diluted with human plasma to 10^5^ GE/ml. Two further plasma samples were prepared as specificity controls, one of them containing yellow fever virus [YF (YF-17D strain)], West Nile virus [WN (New York)], Japanese encephalitis virus [JE (strain SA-14-02)] and tick-borne encephalitis virus [TBE (strain Absettarov)], and a second one containing chikungunya virus [CHIK (strain Marseille LR 2006/684-1)], a common differential diagnosis of DF. A negative control plasma sample was also included (Table 1). Aliquots of 100 µl were number-coded, freeze dried for 24 h (Christ, AlphaI-5, Hanau, Germany) and stored at 4°C until dispatch.

**Table 1:** Results of the EQA for the molecular detection of DENV.

		Samples no.													
		#2	#9	#12	#4	#14	#5	#13	#6	#10	#11	#3	#7		
		DENV-1	DENV-1	DENV-1	DENV-1	DENV-1	DENV-3	DENV-3	DENV-2	DENV-4	JE/YF WN/TBE	CHIK	Negative		
		Copy no. [GE/mL]													
Lab. no.	RT-PCR technique	7,0E+05	7,0E+04	7,5E+03	7,0E+02	7,0E+01	3,0E+04	3,0E+03	1,0E+05	1,0E+05	neg.	neg.	neg.	Score*	Classification #
8	Heminested[17]	++	++	++	+ +	++	++	++	++	++	−	−	−	22	**OPTIMAL**
7	TaqMan[12]	++	++	++	++	*(−)*	++	++	++	++	−	−	−	22	**OPTIMAL**
13	SYBR-Green[18]	++	++	++	++	*(−)*	++	++	++	++	−	−	−	22	**OPTIMAL**
17a	TaqMan[14]	++	++	++	++	*(−)*	++	++	++	++	−	−	−	22	**OPTIMAL**
12	TaqMan[19]	++	++	++	+	++	+	*(−)*	++	++	−	−	−	20	IMPROVE
21	SYBR-Green^a^	++	++	++	++	*(−)*	++	++	++	++	**(+)**	−	−	20	IMPROVE
2a	Nested[12]	++	++	++	*(−)*	*(−)*	++	++	++	++	−	−	−	20	**OPTIMAL**
2b	TaqMan^a^	++	++	++	*(−)*	*(−)*	++	*(−)*	++	++	−	−	−	18	IMPROVE
4b	Nested[28]	++	++	++	*(−)*	*(−)*	++	*(−)*	++	++	−	−	−	18	IMPROVE
28a	Nested^b^	++	++	++	*(−)*	*(−)*	++	*(−)*	++	++	−	−	−	18	IMPROVE
15	TaqMan[20]	++	++	*(−)*	*(−)*	++	++	*(−)*	++	++	−	−	−	18	IMPROVE
5	TaqMan[21]	++	++	++	++	*(*−*)*	*(*−*)*	*(*−*)*	++	++	−	−	−	18	IMPROVE
20	TaqMan[21]	++	++	++	++	*(*−*)*	*(*−*)*	*(*−*)*	++	++	−	−	−	18	IMPROVE
14	Nested[12]	+	+	+	+	*(*−*)*	++	++	++	+	−	−	−	17	**ACCEPTABLE**
27	Nested^b^	+	++	++	++	*(*−*)*	++	*(*−*)*	++	++	**(+)**	−	−	17	IMPROVE
28b	TaqMan^a^	++	++	*(*−*)*	*(*−*)*	*(*−*)*	++	*(*−*)*	++	++	−	−	−	16	IMPROVE
29	TaqMan[18]	++	++	++	*(*−*)*	*(*−*)*	++	*(*−*)*	++	*(*−*)*	−	−	−	16	IMPROVE
31	TaqMan^a^	+	+	+	+	+	+	+	+	+	−	−	−	15	**ACCEPTABLE**
23b	TaqMan^a^	+	+	*(*−*)*	*(*−*)*	*(*−*)*	++	*(*−*)*	++	++	−	−	−	14	IMPROVE
19a	Nested[14]	++	++	*(*−*)*	*(*−*)*	*(*−*)*	++	*(*−*)*	*(*−*)*	++	−	−	−	14	IMPROVE
1	TaqMan^c^	++	++	*(*−*)*	*(*−*)*	*(*−*)*	+	+	++	*(*−*)*	−	−	−	14	IMPROVE
36	Nested[28]	+	+	+	*(*−*)*	*(*−*)*	+	+	+	+	−	−	−	13	**ACCEPTABLE**
10	TaqMan[17]	+	+	+	*(*−*)*	*(*−*)*	+	+	+	+	−	−	−	13	**ACCEPTABLE**
19b	TaqMan[19]	+	+	+	*(*−*)*	+	+	+	+	*(*−*)*	−	−	−	13	IMPROVE
25b	Nested[15]	++	+	*(*−*)*	*(*−*)*	*(*−*)*	*++*	*(*−*)*	++	*(*−*)*	−	−	−	12	IMPROVE
9a	Nested^b^	++	++	++	*(*−*)*	*(*−*)*	++	++	++	*(*−*)*	**(+)**	−	−	12	IMPROVE
9b	TaqMan^a^	+	+	+	+	*(*−*)*	+	+	+	+	**(+)**	−	−	12	IMPROVE
22	TaqMan[13]	++	++	*(*−*)*	*(*−*)*	*(*−*)*	*(*−*)*	*(*−*)*	*(*−*)*	++	−	−	−	12	IMPROVE
4a	TaqMan^a^	+	+	+	*(*−*)*	*(*−*)*	+	*(*−*)*	+	+	−	−	−	12	IMPROVE
30	Nested[28]	*(*−*)*	++	*(*−*)*	++	++	*(*−*)*	*(*−*)*	+	*(*−*)*	−	**(+)**	−	11	IMPROVE
17b	TaqMan[19]	+	+	*(*−*)*	*(*−*)*	+	*(*−*)*	+	*(*−*)*	*(*−*)*	−	−	−	11	IMPROVE
37	TaqMan[22]	+	+	*(*−*)*	*(*−*)*	*(*−*)*	+	+	+	*(*−*)*	−	−	−	11	IMPROVE
3	SYBR-Green^a^	+	*(*−*)*	*(*−*)*	*(*−*)*	*(*−*)*	+	*(*−*)*	+	+	−	−	−	10	IMPROVE
16	TaqMan^c^	++	+	*(*−*)*	++	+	+	+	+	+	**(+)**	**(+)**	**(+)**	10	IMPROVE
18	TaqMan[19]	+	+	*(*−*)*	*(*−*)*	*(*−*)*	*(*−*)*	*(*−*)*	+	+	−	−	−	10	IMPROVE
24	RT-PCR^b^	+	+	*(*−*)*	*(*−*)*	*(*−*)*	*(*−*)*	*(*−*)*	+	+	−	−	−	10	IMPROVE
6	TaqMan^c^	+	+	*(*−*)*	*(*−*)*	*(*−*)*	+	*(*−*)*	+	*(*−*)*	−	−	−	10	IMPROVE
25a	TaqMan[19]	+	+	*(*−*)*	*(*−*)*	*(*−*)*	+	*(*−*)*	+	+	−	−	−	10	IMPROVE
11	Nested[15]	++	++	*(*−*)*	*(*−*)*	*(*−*)*	+	*(*−*)*	+	*(*−*)*	**(+)**	−	−	10	IMPROVE
35a	Nested[12]	++	++	*(*−*)*	*(*−*)*	*(*−*)*	*(*−*)*	*(*−*)*	*(*−*)*	*(*−*)*	−	−	−	10	IMPROVE
34	TaqMan[23]	+	+	*(*−*)*	*(*−*)*	*(*−*)*	*(*−*)*	*(*−*)*	*(*−*)*	+	−	−	−	9	IMPROVE
23a	SYBR-Green[29]	+	*(*−*)*	*(*−*)*	*(*−*)*	*(*−*)*	*(*−*)*	*(*−*)*	+	*(*−*)*	−	−	−	8	IMPROVE
32	Heminested[17]	++	*(*−*)*	*(*−*)*	*(*−*)*	*(*−*)*	*(*−*)*	*(*−*)*	*(*−*)*	*(*−*)*	−	−	−	8	IMPROVE
33	TaqMan[19]	+	+	+	*(*−*)*	*(*−*)*	*(*−*)*	*(*−*)*	+	*+*	−	−	−	8	IMPROVE
26	Nested^b^	*(*−*)*	*(*−*)*	*(*−*)*	*(*−*)*	*(*−*)*	*(*−*)*	*(*−*)*	*(*−*)*	*(*−*)*	−	−	−	6	IMPROVE
35b	Nested[16]	*(*−*)*	*(*−*)*	*(*−*)*	*(*−*)*	*(*−*)*	*(*−*)*	*(*−*)*	*(*−*)*	+	−	−	**(+)**	5	IMPROVE
Correct positive/total results (%)		43/46 (93.5)	41/46 (89)	23/46 (50)	14/46 (30.4)	8/46 (17.4)	32/46 (69.5)	17/46 (37)	38/46 (82.6)	32/46 (69.5)	40/46 (87)	44/46 (95.6)	44/46 (95.6)		

*The score reflects the quality of the diagnostic results: ++, positive result and correctly typed (score 2 points); +, positive but no information on serotype or incorrectly typed (score 1 point); **(+)**, false positive (score 0 points); −, correct negative result (score 2 points); *(*−*)*, false negative (score 0 points). Sample #14 was not evaluated for scoring.

# Classification reflects the capability to perform optimal surveillance and diagnosis activities (sensitivity up to 10^3^ GE/ml and serotyping); acceptability for diagnosis purposes (sensitivity up to 10^3^ GE/ml); or the need of improvement (failure to detect one or more serotypes or sensitivity below 10^3^ GE/ml). Samples #4 and #14 were not evaluated for classification.

***a*** In-house qRT-PCR system(s) for quantification and/or serotyping;

***b*** In-house RT-PCR system(s);

***c*** Commercial Artus Light Cycler test (Qiagen).

Four different sets of test aliquots were tested before distribution by the Robert Koch Institute (RKI), Berlin, Germany. After reconstitution with 100 µl of water, samples were extracted using the QIAamp viral RNA minikit (Qiagen, Hilden, Germany) according to the manufacturer's instructions. DENV genome copies present in the samples were estimated by an in-house real time PCR and serotypes were confirmed by nested RT-PCR and sequencing [11]. Additionally, the sample panel was analysed independently by one expert laboratory (USAMC-AFRIMS, Bangkok, Thailand) using an RT-PCR assay[12] and an in-house quantitative real time RT-PCR. This laboratory was blinded to sample contents before analysis.

A temperature/time-stability control of the EQA samples was additionally performed at the RKI. A set of samples were placed at room temperature for 1 month, analysed for maintenance of their properties, and compared to a set of samples stored at 4°C.

The EQA panel was distributed to all participants with full instructions. Samples were shipped at ambient temperature by post to participating laboratories. Participants were requested to resuspend the samples in 100 µl of water and to analyse the material as serum samples for DENV molecular diagnosis. They were requested to report their results, and any problems encountered, as well as to provide information on the assay details (RT-PCR method and extraction procedure) using a common form included in the documentation.

### Evaluation of the results

To assure anonymous participation, an individual numerical identification code was assigned to the results sent by each laboratory. This number was followed by a letter (a, b) when different sets of results (more than one method) were sent.

The results were scored in reflection of sensitivity, specificity, and correct serotyping. Two points were given for correct dengue-positive results and correct serotyping. One point was given for correct dengue-positive results without or incorrect type determination. False-negative or false-positive results were scored with no points. Sample #14 (DENV-1; 70 GE/ml) was not considered for scoring. Quantitative data were considered only informative about the availability of this data.

Additionally, results were classified as optimal for diagnosis and surveillance (full detection up to 10^3^ GE/ml *and* serotyping results), acceptable for diagnosis (full detection up to 10^3^ GE/ml), or need of improvement (failure to detect one or more DENV serotypes or sensitivity below 10^3^ GE/ml). Samples #4 (DENV1; 7E+02 GE/ml) and #14 (DENV1; 70 GE/ml) were not considered for classification. No false-positive results were allowed in virus-free samples.

Test results were sent out in an anonymous manner to all participants.

### Statistical analysis

Data collected were entered into Microsoft Excel (Microsoft Corp., Bellingham, WA, USA) and analysed using SPSS 14.0 for Windows. To project performance of a hypothetical average laboratory, cumulative fractions of positive results reported for each test sample of the 10-fold dilution series with DENV-1 were correlated against RNA concentrations in samples and subjected to probit analysis. Results with respect to categorised variables were analysed by the Chi-square test. Parametric (independent sample t-test and ANOVA) and non-parametric (Mann-Whitney and Kruskall-Wallis) tests were used to estimate the influence of the PCR format on the performance.

## Results

Sixty-seven institutions involved in laboratory diagnostics of DENV infection were invited to participate in this study. Invitees were members of the International WHO DengueNet, the ENIVD-CLRN, or national/regional dengue reference laboratories. The study was announced as an EQA study on DENV molecular diagnostic proficiency, which included certifying and publishing the results in a comparative and anonymous manner.

Thirty-seven laboratories (response rate 55.2%) from 27 countries participated in the study. A total of 46 sets of results were received (Table 1), including 9 double sets from laboratories that used 2 methods each (sets 2ab, 4ab, 9ab, 17ab, 19ab, 23ab, 25ab, 28ab, 35ab).

A variety of tests were used for screening and identification of DENV by participating laboratories; these included nested (n = 15, 32.6%)[11], [12], [13], [14], [15], [16] and hemi-nested (n = 2, 4.3%) RT-PCR [17], TaqMan (n = 25, 54.3%) [13], [14], [18], [19], [20], [21], [22], [23] and SYBR Green (n = 4, 8.7%)-based real time PCR (Table 2).

**Table 2:** Probit analysis of the sensitivity profile of the different RT-PCR methods reported.

Method used for RT-PCR	Protocols	Percentage	Low sensitivity#	Positive performance*50% [CI 95%]	Positive performance*95% [CI 95%]
**RT nested PCR**	[*12*], [*14*], [*11*], *l * [*13*], [*15*], [*16*],*a*	32.6% (15/46)	80% (12/15)	10^4^ GE/ml[3×10^3^−4.2×10^4^ GE/ml]	4×10^6^ GE/ml[5.7×10^5^−3.6×10^8^ GE/ml]
**RT heminested PCR**	[*17*]	4.3% (2/46)	50% (1/2)	NP	NP
**TaqMan** **real time PCR**	[*12*],[[*14*], [*18*], [*14*], [*19*]–[*20*], [*21*], [*13*],[*22*],[*23*],*a; b*	54.3% (25/46)	76% (19/25)	10^4^GE/ml[2.8×10^3^−4×10^4^ GE/ml]	4.2×10^6^GE/ml[5.2×10^5^−4.4×10^8^GE/ml]
**SYBR Green** **real time PCR**	[*18*], [*29*],*a*	8.6% (4/46)	50% (2/4)	6.3×10^4^GE/ml.[NP]	3×10^5^ GE/ml[NP]

NP: Not enough data for statistical analysis; *a*: in-house protocol; *b*: Commercial kit;

*Probit analysis.

# Sensitivity below 10^3^ GE/ml.

Data fit into the model with p<0.05.

Different RNA extraction methods were reported by the participating laboratories; QIAmp Viral RNA Minikit (QIAGEN) [n = 19, 51.4%], EZ1 Biorobot (QIAGEN) [n = 2, 5.4%], QIAmp UltraSens kit (QIAGEN) [n = 1, 2.7%], RNeasy kit (QIAGEN) [n = 1, 2.7%], QIASymphony Viral RNA extraction kit (QIAGEN) [n = 1, 2.7%], Macherey Nagel (Düren, Germany) RNA virus kit [n = 1, 2.7%], NucliSens Easy Mag (BIOMERIEUX, Nürtingen, Germany) [n = 1, 2.7%]. Unfortunately, eleven laboratories (29.7%) did not report data on the extraction procedure.

Performance varied among the laboratories according to whether the methodologies used achieved a full or acceptable result (Table 1). Only 5 out of 46 (10.9%) analyses reported met all criteria with optimal performance; 4 out of 46 (8.7%) data sets achieved acceptable results; and 37 out of 46 (80.4%) of the results reported showed that there is room for improvement regarding DENV diagnosis. In 22 cases non-optimal results were due to the failure to identify one or more DENV serotypes: DENV-2 (lab n°19a); DENV-3 (n° 5, 20, 18, 24, 33); DENV-4 (n° 29, 1, 19b, 25b, 9a, 37, 6, 11); DENV2 and DENV3 (n° 22, 34); DENV2 and DENV4 (n° 17b); DENV-3 and DENV-4 (n° 30, 23a); DENV-2, DENV-3 and DENV-4 (n° 35a, 32); DENV-1, DENV-3 and DENV-2 (n° 35b); one laboratory completely failed in the detection of positive DENV samples (n° 26). Failures due to the presence of false positives were present in 8 data sets (n° 21, 9a, 9b 27, 30, 16, 11, 35b).

The serial dilutions of DENV-1 (samples #2, #9, #12, #4, and #14) and DENV-3 (samples #5 and #13) showed a clear tendency of low sensitivity with increased dilution, this being the major reason for non-acceptable results (Table 1). Samples #4 and #14 (700 and 70 GE/ml, respectively) were not considered in the classification, assuming that a minimal detection of 7,000 GE/ml should be achieved to perform a reliable DENV molecular diagnosis. Only 47.7% (n = 22) of the entries reported positive results in detecting 7,000 GE/ml of DENV-1, and 36.3% (n = 17) of the data sets achieved a positive result in the 3,000 GE/ml DENV-3 samples.

**Figure 1:**
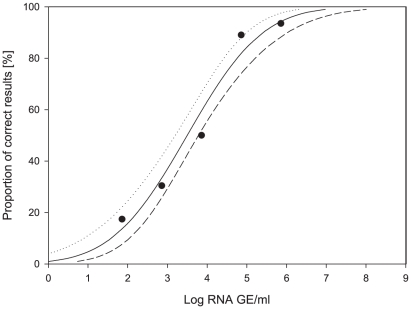
Probit analysis of data sets with a correct result (y axis) for DENV-1 related to viral RNA concentration in positive samples (x axis). Data points represent individual samples in the panel. The thick line is the regression line calculated on the basis of a probit model (dose-response curve), and thin lines are 95% confidence intervals. Data fit into the model with p<0.003.

A probit analysis was performed using positive results reported for each sample of the 10-fold DENV-1 dilution series correlating with RNA concentrations in samples (Figure 1). The data demonstrated that globally 50% of positive performance could be expected in concentrations higher than 10^4^ GE/ml (95% confidence interval [CI] 23,110–32,000 GE/ml). A certainty of 95% was achieved for 5×10^4^ GE/ml (95% CI 41,760–57,500 GE/ml). This pointed to the need of improvement of the laboratories' capacity for achieving positive results in lower viral loads, so the existence of false negatives in routine practice of DENV diagnosis could be minimized.

A probit analysis of the correct results obtained, using TaqMan-based real time PCR and RT nested PCR protocols, showed that 50% of positive performance could be expected when viral genome concentration in the sample is higher than 10^4^ GE/ml (Table 2). 95% of certainty was achieved with TaqMan real time PCR and RT nested PCR when more than 4.2×10^6^ GE/ml (95% CI [5.2×10^5^–4×10^8^]) or 4×10^6^ GE/ml (95% CI [5.7×10^5^–3.6×10^8^]), respectively were present in the sample (Table 2). T-test comparing the TaqMan real time performance versus RT nested PCR and SYBR Green real time RT-PCR did not reveal any significant difference in performance for the applied methods. In general, the choice of the real-time PCR format seemed to have no effect on the success of the analysis which is mostly affected by the performance of the individual laboratories as demonstrated by the wide range of the confidence intervals.

As many as 9 laboratories (n° 2, 17, 28, 19, 9, 4, 25, 35, and 23) used two different RT-PCR techniques for the evaluation. This approach is advantageous to exclude false positives/negatives in routine diagnosis. One laboratory used two different TaqMan PCRs (17ab), one laboratory used a combination of TaqMan- and SYBR Green-based PCRs (23a,b), one laboratory used two different RT nested PCR techniques (35a,b); and six laboratories used a combined approach of TaqMan real time PCR and RT nested PCR (2a,b; 19a,b; 25a,b; 9a,b; 28a,b; 4a,b).

Only 26 data sets (56.5%) included serotyping results that are necessary for achieving optimal results and support the reliability of the techniques and the laboratories to perform both diagnosis and surveillance activities.

Further information on the number of copies of DENV strains referred to the participants was requested to estimate the experience in viral load determination in DENV diagnosis. Only 8 laboratories out of 37 (21.6%) reported quantitative results (data not shown), even though as many as 29 laboratories (78.4%) were using real time-based procedures which would have allowed obtaining this information.

Results reported from different laboratories using the same techniques differed in their final performance, probably due to differences in the individual operational procedures and not to limitations of the methods themselves (Table 1). This is the case of 6 laboratories which reported data obtained using the TaqMan PCR developed by Drosten et al[19], where a different competence was observed depending on the reporting laboratory. On the other hand, in some cases, the same limitations were observed in laboratories using a common technique, thus indicating that an improvement or up-dating of the technique might be needed. This is the case of those laboratories using a TaqMan PCR from Leparc-Gofart et al[21] which failed to detect DENV-3 (Table 1).

A stability assessment of the samples when stored at room temperature (23–25°C) for 1 month (Table 3) demonstrated that the failure in detecting the DENV RNA experienced by some laboratories was probably not due to genome degradation caused by shipment conditions.

**Table 3:** Stability of the lyophilized panel of samples.

#2	#9	#12	#4	#14	#5	#13	#6	#10		
DEN-1	DEN-1	DEN-1	DEN-1	DEN-1	DEN-3	DEN-3	DEN-2	DEN-4		
7×10^5^	7×10^4^	7×10^4^	NEG	NEG	2×10^4^	1×10^3^	6×10^4^	1×10^5^	GE/ml	4°C
4×10^4^	1.4×10^4^	1×10^3^	90	NEG	1.5×10^4^	2×10^3^	7.3×10^4^	1×10^5^	GE/ml	RT

A set of YF EQA samples were kept at room temperature (23–25°C) for 1 month. These samples were analysed in parallel with a control set of samples kept at 4°C using an in-house real-time RT-PCR to control the level of stability/degradation of the samples when sent by mail at room temperature.

Some non-optimal results were due to the presence of false positives even when a good profile of sensitivity and thus a high score was reached (n° 21, 9b). False positives were mostly found on sample #11, containing other flaviviruses like Japanese encephalitis, yellow fever, West Nile and tick-borne encephalitis virus.

Of 13 laboratories taking part in the first DENV molecular diagnosis EQA realised in 2003[9], eight laboratories participated in the present EQA. Four laboratories improved their percentage of correct results, three experienced a decrease, and 1 laboratory reached 100% of correct results in both EQAs (data not shown).

## Discussion

EQA studies for laboratory molecular diagnostics of DENV help to monitor the quality and accuracy of current diagnosis and the capacity for surveillance of the participating centres, to highlight problems in particular techniques or specific laboratories, and to give advice and assurance to all the centres. This is the second DENV molecular diagnosis EQA under the auspices of the ENIVD-CLRN project[9].

Twenty-two laboratories out of 37 (59.4%) participants were located in Europe, a non-endemic area. Even though the EQA was announced widely to national and international public health institutions involved in dengue control and surveillance in endemic areas, the number of responses was not as abundant as expected. Among the reasons for that, we should consider the cost of the reagents to be consumed for the exercise and to be covered by the individual laboratories, or the unwillingness to send incorrect results. Even though, it has to be considered that laboratories in endemic countries, that may not have participated in this EQA but perform dengue molecular diagnosis routinely, usually have strong internal quality control programs, and there are numerous laboratories that do perform internal QC procedures. Participation in EQA diagnosis activities must be encouraged and supported by health institutions as they provide helpful information on the capabilities of the health system to detect and control the disease.

In the present study the performance of molecular methods for DENV diagnosis with different designs varies depending on the method and the reporting laboratory. It is difficult to assess which methods are better, since primers, enzymes, buffers, reaction conditions, genome target, and thermocyclers can all influence the reliability of a PCR[24]. However, some conclusions could be assumed from the results obtained in this EQA.

Surprisingly, a low sensitivity was appreciated in the general results of this EQA, even when reliable real time procedures were used. We selected a cut-off of ≥10^3^ GE/ml to achieve an optimal or acceptable classification because it is considered over the above limit of detection of current RT-PCR protocols [6]. Secondly, we have also assumed this to be a suitable detection threshold for diagnosis of acute dengue according to published results on viral load titers in DF and DHF patients [25], [26], [27]. Extreme precaution should be taken when performing molecular diagnosis using methods with a limited sensitivity, so it could suppose the presence of false negatives in the diagnostic results. Likewise, in the final classification, no false positives results were allowed in virus-free samples, and the inability to detect one or more DENV serotypes was also considered non-acceptable, as this could lead to an incorrect DENV diagnosis with high impact on the clinical and public health management of DENV cases. In our study, false positives were found mainly in sample #11, which contained viral genome of different flaviviruses (Japanese encephalitis virus, tick-borne encephalitis virus, West Nile virus, and yellow fever virus). This reflects the need for further improvement on the specificity of the methodologies and/or the procedures carried out in the laboratories to avoid cross-contamination.

The sets of samples were sent by post to the participating laboratories, and it was of concern in some cases that it took long for them to reach their destination while maintained at ambient temperature. We tested the stability of the samples when stored at room temperature and demonstrated that genome degradation caused by shipment conditions was minimal.

In this study, a clear significant difference in sensitivity could not be noted when real time RT-PCR procedures were compared to RT-nested or heminested-PCR protocols, so no conclusions can be ventured in this regard. It should be said, however, that the most important factor in the reliability of the results for dengue diagnosis is the individual performance of each laboratory, as demonstrated by the different score values reached by different laboratories that used the same published protocols. This should alert to the need to revise the running protocols and processes in those laboratories without good results.

Different extraction methods have been reported, with most of the laboratories using QIAGEN kits for viral RNA extraction. Unfortunately, we did not receive this information from all laboratories, so we could not perform an accurate statistical analysis. However, in previous EQAs, the extraction procedures showed no great relevancy regarding final performance[9].

Discrepancies due to the reporting of an incorrect serotype were considered acceptable for diagnosis, since the case management would not have been affected. However, the absence of serotyping procedures or non-reliable ones diminished the possibility of performing surveillance studies. In those laboratories in non-endemic areas where dengue is diagnosed in the context of an “imported” disease (i.e. European laboratories), serotype classification of the cases could be considered unnecessary for clinical management of the patients, whereas in DENV-endemic countries it is highly desirable for reference laboratories to contribute serotype information, since established surveillance systems should monitor where DENV is transmitted, which serotypes are involved, and which type of illness is associated with these serotypes.

Quantification of DENV RNA in human plasma samples can provide more clues for performing pathogenesis studies and monitoring the progress of clinical manifestations [25], [26], [27]. The use of different quantitative real time PCRs has been reported on this EQA, and 29 different data sets were obtained using these techniques. However, the absence of quantification data in most of participating laboratories is remarkable.

In order to improve awareness and technical performance of DENV reference laboratories, we recommend the continuation of these EQA exercises.

## Supporting Information

Checklist S1 STARD Checklist
(0.98 MB PDF)
